# GeneBins: a database for classifying gene expression data, with application to plant genome arrays

**DOI:** 10.1186/1471-2105-8-87

**Published:** 2007-03-12

**Authors:** Nicolas Goffard, Georg Weiller

**Affiliations:** 1 ARC Centre of Excellence for Integrative Legume Research and Bioinformatics Laboratory, Genomic Interactions Group, Research School of Biological Sciences, Australian National University, GPO Box 475, Canberra, ACT 2601, Australia

## Abstract

**Background:**

To interpret microarray experiments, several ontological analysis tools have been developed. However, current tools are limited to specific organisms.

**Results:**

We developed a bioinformatics system to assign the probe set sequences of any organism to a hierarchical functional classification modelled on KEGG ontology. The GeneBins database currently supports the functional classification of expression data from four Affymetrix arrays; *Arabidopsis thaliana*, *Oryza sativa, Glycine max *and *Medicago truncatula*. An online analysis tool to identify relevant functions is also provided.

**Conclusion:**

GeneBins provides resources to interpret gene expression results from microarray experiments. It is available at

## Background

Microarrays enable us to study the expression of thousands of genes simultaneously, providing a comprehensive overview of the gene activities in a given tissue. A number of ontological tools are now available that support the functional interpretation of gene expression data, through the identification of significant enriched Gene Ontology terms (GO) [1] associated with a list of (differentially expressed) genes, such as Onto-Tools [2], BlastSets [3], NetAffx [4], ArrayXPath [5] or FatiGO [6]. However, Gene Ontology is a controlled vocabulary designed to organize information for molecular function, biological processes and cellular components and thus does not directly reflect metabolic pathways. In addition, these tools are limited to organisms with well-annotated genomes.

We propose a new strategy that assigns genes to hierarchical categories (BINs) modelled on the ontology provided by the KEGG database [7]. KEGG is a pathway-orientated database, which integrates the genes of many species. The top level of the classification contains four categories (metabolism, genetic information processing, environmental formation processing and cellular processes); the next levels correspond to subcategories (e.g. metabolic pathways, multiprotein complexes, protein families, etc.) or to individual functions. By converting the entire KEGG Orthologous database into a new BIN structure (GeneBins), we define a generic hierarchical classification (i.e. not species-specific). Any protein gene can then be assigned to a bin in this ontology based on the similarity of its amino acid sequence to the sequences in four reference databases (KEGG, Cluster of Orthologous Groups (COG) [8], Swiss-Prot [9] and Gene Ontology), using the cross-references provided by KEGG. Based on this approach, GeneBins currently contains probe set assignments to the KEGG-based ontology for the Affymetrix arrays [10] of *Arabidopsis thaliana, Oryza sativa *(rice) and the model legumes *Glycine max *(soybean) and *Medicago truncatula *(barrel medic).

Based on these assignments, we have developed an online tool to identify the significantly over- or under-represented metabolic pathways in a set of sequences using a method based on the hypergeometric distribution, as developed in the BlastSets system [3]. This can, for example, be used to interpret sets of up- or down-regulated microarray sequences.

In addition, the classification system provided can also be used in MapMan [11-13] to display gene expression data on images representing a functional context of these genes, for which it provides both the BIN structure and mapping file to this ontology.

## Construction and contents

The GeneBins database is a web-based tool combining a PostgreSQL database management system with a dynamic web interface based on PHP and Perl. Data pre-processing is implemented in Perl and statistical analyses are performed using Perl and the R statistical package [14].

The database contains three components:

i. The functional hierarchy (GeneBins structure) consists of two tables; the first table contains the identifiers (BIN codes) and their descriptions (BIN names) and the second contains the hierarchical structure of the classification.

ii. The reference databases with identifiers, description and protein sequences from KEGG Orthologous, COG, Swiss-Prot and the reference set of sequences provided by Gene Ontology.

iii. The genome arrays containing data from the Affymetrix arrays. Each probe set is described by its identifier, the database from which the sequence used to design the probe set was taken, the accession number and description of a representative sequence, and the consensus sequence spanning from the most 5' to the most 3' probe position in the public Unigene cluster.

Probe sets are assigned to the GeneBins hierarchy based on their sequence similarity with amino acid sequences in the reference databases. BINs are linked to these sequences by the cross-references provided by KEGG. We used BLASTX [15] to find best matches (E-value < 10^-8^) for each consensus sequence of a given Affymetrix array in each reference database. From these we extracted cross-references to assign the probe set to the corresponding BIN in the GeneBins classification.

As of August 2006, data for the Affymetrix arrays of four plants (*Arabidopsis thaliana*, *Oryza sativa, Glycine max *and *Medicago truncatula*) are available in the database (Table 1).

**Table 1:** Affymetrix arrays available and assignment statistics

			**Unclassified**	
**Affymetrix array**	**Sequences**^1^	**Classified**^2^	**Homolog**^3^	**No homolog**^4^
Arabidopsis ATH1 Genome Array	30,193	9,520	17,787	2,886
Rice Genome Array	57,194	15,023	16,859	25,312
Soybean Genome Array	37,618	9,842	13,286	14,490
Medicago Genome Array	50,900	13,322	15,990	21,588

^1^Number of probe set sequences

^2^Number of probe set sequences that have been assigned to classified BINs

^3^Number of probe set sequences not classified but homologs for these sequences have been found

^4^Number of probe set sequences not classified and without homolog in reference databases

## Utility and discussion

The GeneBins web interface [16] can be used to search the classification of a given probe set or to analyse a list of identifiers according to their assignments in the hierarchy.

### Search for classification

**Figure 1:**
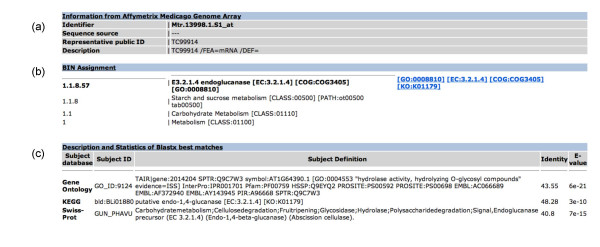
**Screenshot of search results for the probe set Mtr.13998.1.S1_at in the Affymetrix Medicago Genome Array**. This page shows: (**a**) information from the genome array, the database from which the sequence used to design this probe was taken, the accession number of a representative sequence and the associated description; (**b**) BIN assignment of the submitted probe set and its position in the hierarchy; (**c**) description and statistics of BLASTX best matches used to classify the probe set.

It is possible to retrieve the classification of a probe set in a selected genome array by its Affymetrix probe set identifier or by the GenBank accession number of the representative sequence. The results of database queries provide information on the probe set sequence, its position in the functional hierarchy, and the blast matches, as given in Figure 1. Note that a probe set can be assigned to more than one BIN. The cross-references associated to these BINs are displayed with a hyperlink to the entry in the corresponding database. The best BLAST matches are used to assign the probe set sequence to the BINs, provided that they exceed a pre-defined threshold E-value (10^-8^).

### Gene expression analysis

GeneBins can be used to identify the functional categories associated with a set of sequences (e.g. differentially expressed) and thus find the metabolic pathways or other cellular functions up- or down-regulated in microarray experiments. The list of probe set identifiers (Affymetrix probe set identifiers and/or GenBank accession numbers), belonging to a given genome array, can be pasted in a text box or uploaded from a file in the GeneBins website.

**Figure 2:**
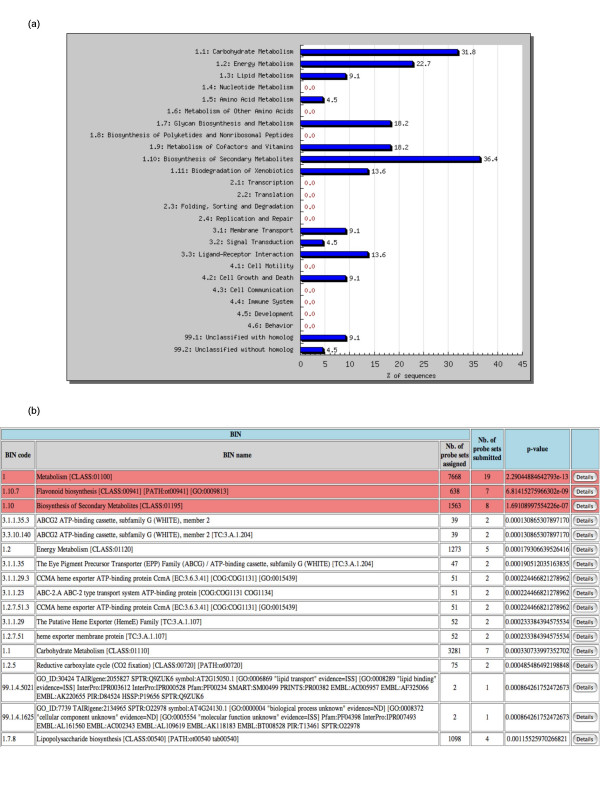
**Screenshots of a gene list analysis**. (**a**) Example of a functional distribution of a list of submitted probe sets in the 2nd level of the GeneBins ontology. The percentage represents the proportion of submitted probe sets that have been assigned in the corresponding category (BIN). (**b**) List of BINs that intersect with a list of submitted probe sets. Each row reports information concerning the position in the functional hierarchy (BIN code), its description (BIN name) and the number of probe sets in the BIN. The comparison of groups is reported with: the number of submitted probe sets that fall in the corresponding BIN and the p-value for finding the group by chance, based on the hypergeometric distribution. The significant BINs are highlighted in red. For each BIN, the 'Details' button is linked to the list of all probe sets assigned to this group with the submitted sequences highlighted.

To provide an overview of the functions affected, a bar plot representing the distribution of the submitted identifiers in the second level of the classification is displayed (Figure 2a). Note that the sum of the percentages can be more than 100% as a gene can be assigned to several BINs.

To detect if a certain functional category is statistically over-represented in the selected group of genes, compared to the rest of the genome array, the p-value for all BINs throughout the classification is calculated using the hypergeometric distribution [17]. This p-value represents the probability that the intersection of the set of submitted sequences with the set of sequences belonging to the given BIN occurs by chance. The p-value significant threshold can be specified, with a default cut-off of 0.05. Because multiple hypothesis tests are performed, it can also be adjusted using a Bonferroni correction [18]. The resulting page lists, by increasing p-values, the BINs with assigned probe sets belonging to the submitted group (Figure 2b). Those that are significant are highlighted. It is possible to retrieve the list of all probe sets assigned to a given BIN. This page can be bookmarked as the results are stored for seven days, and can also be downloaded in a tabular file.

In addition, to display gene expression data on images representing a functional context of these genes (e.g. metabolic pathways) using MapMan, the complete probe sets classification for each organism can be downloaded in the appropriate MapMan format and in an xml format to be explored locally using any outliner.

### Future developments

In the near future, we plan to apply our approach to other Affymetrix arrays. The classification process will be improved by taking into account the domain composition of the proteins. We are currently developing an interface allowing the submission of a set of sequences (e.g. custom DNA microarrays) to be classified automatically.

## Conclusion

GeneBins provides a hierarchical functional classification, modelled on the KEGG ontology, of probe set sequences of four plant Affymetrix arrays. Based on these assignments, an online analysis tool is available to interpret gene expression results from microarray experiments by identifying the most relevant pathways or functions involved in a submitted list of genes.

## Availability and requirements

Access to GeneBins is via a web interface, freely available to all interested users, at 

It has been tested to work with Safari 2.0, Mozilla Firefox 1.5 and Internet Explorer 6.0 web browsers and does not require any particular plug-in.

## Authors' contributions

NG participated in the design, implemented the system and drafted the manuscript with revisions provided by GW. GW conceived and supervised the project. Both authors read and approved the final manuscript.
